# A diagnostic index based on pseudo-continuous arterial spin labeling and T1-mapping improves efficacy in discriminating Alzheimer’s disease from normal cognition

**DOI:** 10.3389/fnins.2022.974651

**Published:** 2022-08-05

**Authors:** Xiaonan Wang, Di Wang, Xinyang Li, Wenqi Wang, Ping Gao, Baohui Lou, Josef Pfeuffer, Xianchang Zhang, Jinxia Zhu, Chunmei Li, Min Chen

**Affiliations:** ^1^Department of Radiology, National Center of Gerontology, Beijing Hospital, Institute of Geriatric Medicine, Chinese Academy of Medical Sciences, Beijing, China; ^2^Graduate School of Peking Union Medical College, Chinese Academy of Medical Sciences, Beijing, China; ^3^Department of Radiology, Children’s Hospital, Zhejiang University School of Medicine, Hangzhou, China; ^4^Department of Neurology, National Center of Gerontology, Beijing Hospital, Institute of Geriatric Medicine, Chinese Academy of Medical Sciences, Beijing, China; ^5^MR Application Development, Siemens Healthcare GmbH, Erlangen, Germany; ^6^MR Collaboration, Siemens Healthineers Ltd., Beijing, China

**Keywords:** Alzheimer’s disease, cerebral blood flow, T1-mapping, magnetic resonance imaging, arterial spin labeling

## Abstract

**Background:**

Pseudo-continuous arterial spin labeling (pCASL) is widely used to quantify cerebral blood flow (CBF) abnormalities in patients with Alzheimer’s disease (AD). T1-mapping techniques assess microstructural characteristics in various pathologic changes, but their application in AD remains in the exploratory stage. We hypothesized that combining quantitative CBF and T1 values would generate diagnostic results with higher accuracy than using either method alone in discriminating AD patients from cognitively normal control (NC) subjects.

**Materials and methods:**

A total of 45 patients diagnosed with AD and 33 NC subjects were enrolled, and cognitive assessment was performed for each participant according to the Chinese version of the Mini-Mental State Examination (MMSE). T1-weighted magnetization-prepared 2 rapid acquisition gradient echo (MP2RAGE) and pCASL sequence were scanned on a 3T MR scanner. A brain morphometric analysis was integrated into prototype sequence, providing tissue classification and morphometric segmentation results. Quantitative CBF and T1 values of each brain region were automatically generated inline after data acquisition. Independent samples *t*-test was used to compare regional CBF and T1 values controlled by false discovery rate correction (corrected *p* < 0.01). The model with combined CBF and T1 values was compared with the individual index by performing receiver operating characteristic curves analysis. The associations between the MMSE score and CBF and T1 values of the brain were investigated using partial correlations.

**Results:**

Cerebral blood flow of the right caudate nucleus (RCc) and left hippocampus (LHc) was significantly lower in the AD group compared with the NC group, while the T1 values of the right caudate nucleus (RCt) and left hippocampus (LHt) increased in the AD group. Prediction accuracies of 73.1, 77.2, 75.9, and 81.3% were achieved for each of the above parameters, respectively. In distinguishing patients from controls using the corresponding optimized cut-off values, most combinations of parameters were elevated (area under curve = 0.775–0.894). The highest area under curve value was 0.944, by combining RCc, LHc, RCt, and LHt.

**Conclusion:**

In this preliminary study, the combined model based on pCASL and T1-mapping improved the diagnostic performance of discriminating AD and NC groups. T1-mapping may become a competitive technique for quantitatively measuring pathologic changes in the brain.

## Introduction

Alzheimer’s disease (AD) is a neurodegenerative disease with insidious onset and progressive development. Its main clinical manifestations include memory loss (Jagust et al., 2001), cognitive impairment, and behavioral abnormalities. With the rapidly aging population, degenerative diseases of the central nervous system have become the third most common diseases affecting human survival after cardiovascular and cerebrovascular diseases and cancer (Erkkinen et al., 2018). The number of people with AD-based dementia worldwide is expected to reach 113.4 million by 2050, and the increasing incidence has created a challenging scenario for global public health care systems (Knopman et al., 2021). Despite decades of research, the exact pathogenesis of AD remains to be elucidated. In addition to classical hypotheses, including β-amyloid peptide aggregation leading to neuroplaque formation (Arnold et al., 1991; Montine et al., 2012) and abnormal phosphorylation of tau proteins causing neurogenic fiber tangles (Gallardo and Holtzman, 2019; Dujardin et al., 2020) and early synaptic loss (Rice et al., 2019), multiple risk factors for cerebrovascular disease are closely associated with the development of AD (Cortes-Canteli and Iadecola, 2020). Therefore, discovering non-invasive, objective, quantitative, and reproducible imaging methods that indirectly reflect the pathologic changes of AD pathogenesis will clarify the pathologic process of AD and detect disease progression.

Perfusion abnormality is an integral part of assessing the pathophysiology of AD. Single-photon emission computed tomography (SPECT) and positron emission tomography-computed tomography (PETCT) indirectly reflect blood perfusion through brain glucose metabolism. However, nuclear medicine methods are invasive and involve intravenous radioactive tracer administration, limiting clinical applications. With the development of magnetic resonance imaging (MRI)-based neuroradiology, arterial spin labeling (ASL) is increasingly used as a non-invasive, inexpensive, easily accessible, and reproducible alternative for perfusion measurement (Verfaillie et al., 2015). ASL can quantitatively measure relative cerebral blood flow (CBF) in different brain regions using magnetically labeled water protons in arterial blood as a contrast agent instead of injecting exogenous contrast agents (Chandra et al., 2019). The accuracy of cerebral perfusion maps in AD patients is similar to that of SPECT, whereas ASL is more sensitive to areas with reduced cerebral perfusion (Kaneta et al., 2017). Therefore, this technique may be an alternative to invasive imaging and has the potential to serve as a biomarker in the early diagnosis of preclinical dementia (Xekardaki et al., 2015). Three kinds of ASL techniques are commonly used according to the labeling strategy: continuous ASL, pulsed ASL, and pseudo-continuous ASL (pCASL). Among them, pCASL 3D imaging can be acquired in multiple segments in a short period of time, overcoming magnetic sensitivity and distortion artifacts, and provides good image quality without the need for high-level hardware support (Soman et al., 2021). In this study, we used 3D pCASL to obtain CBF in different brain regions.

Quantitative MRI parameters can reveal not only macroscopic changes in brain tissue but also microstructural changes in the biochemical environment. Among them, longitudinal relaxation time (T1) is the characteristic time governing the relaxation of longitudinal magnetization toward thermal equilibrium after excitation by an RF pulse, and different biological tissues have specific T1 values due to differences in their cellular and interstitial components. Previous studies have shown that an altered T1 value is associated with increased β-amyloid load in the brain of AD patients (House et al., 2008). Experiments based on animal models of AD have also confirmed such tissue alterations (Forster et al., 2013). Previous study found that T1 in the hippocampus, thalamus and right caudate nucleus increased significantly with disease progression in AD patients bilaterally (Su et al., 2016). Therefore, we introduced the T1 values to enhance diagnostic confidence.

We hypothesized that combining CBF and T1 values would yield diagnostic results with greater accuracy than using separate methods in differentiating patients with AD from subjects with normal cognition. To test this hypothesis, we aimed to assess the efficacy of CBF, T1 values, and their combination in discriminating between AD and normal control (NC) cohorts.

## Materials and methods

### Participants

From September 2020 to October 2021, we prospectively recruited patients with AD and cognitively NC subjects from Beijing Hospital. This study was approved by the Ethics Committee of Beijing Hospital (batch no. 2018BJYYEC-147-02 and 2021BJYYEC-123-01), and all participants signed an informed consent form before the examination.

Criteria for inclusion in the AD group were as follows: (1) The neurologists or psychiatrists diagnosed the patients according to the National Institute of Neurological and Communicative Disorders and Stroke/Alzheimer’s Disease and Related Disorders Association(NINCDS-ADRDA) criteria (McKhann et al., 1984). (2) MRI was completed within 48 h after the clinical diagnosis of AD. (3) Right handedness.

Normal control subjects were recruited from 20 community social centers in four districts and were included in this study based on the following criteria: (1) age and gender matching that of the AD group; (2) Chinese version of the MMSE score ≥ 27; and (3) Right handedness.

Exclusion criteria for the AD were as follows: (1) diagnosis of central nervous system tumor (*n* = 1), stroke (*n* = 2), and hydrocephalus (*n* = 1) on routine head MRI examination; (2) dementia of vascular origin with a score of ≥4 on the Harkinski Ischemic Index Scale (*n* = 2); (3) thyroid dysfunction, depression, drug addiction, substance abuse, or other diseases that cause abnormal cognitive function; (4) other reasons that prevented head MRI examination or cooperation with completion of the neuropsychologic scale, Claustrophobia diseases (*n* = 1); and (5) motion and metal artifacts (*n* = 3). Finally, 45 patients with AD and 33 NC individuals were eligible for this study.

### MRI protocol

Clinical information collection and neuropsychologic scale scoring were completed at the Department of Neurology, Beijing Hospital. MRI examinations were performed within 60 min of clinical information collection.

All MR examinations were performed on a 3T MR system (MAGNETOM Prisma, Siemens Healthcare, Erlangen, Germany) with a 64-channel head coil. pCASL was performed using a prototype 3D gradient and spin-echo (GRASE) sequence with the following parameters: TR/TE = 4350/20.9 ms, FOV = 220 mm × 220 mm, matrix = 64 × 64 (interpolated to 128 × 128), bolus duration = 1,800 ms, 16 label timesTI = 800–3800 ms (Δ = 200 ms), and acquisition time = 4 min 55 s including a M0 calibration volume. Regional CBF maps were automatically generated inline after data acquisition (Buxton et al., 1998; Alsop et al., 2015). For quantification, the following parameters were used: blood/tissue water partition coefficient lambda = 0.9 mL/g, labeling efficiency alpha = 60% (four background suppression pulses accounted for), T1_blood = 1650 ms and T1_tissue = 1330 ms. T1 mapping was obtained using a prototype T1 magnetization prepared 2 rapid acquisition gradient echoes (MP2RAGE) sequence with the following parameters: TR/TE = 5000/3.59 ms, TI = 700/2500 ms, FA = 4°/5°, FOV = 230 mm × 216 mm, matrix = 320 × 240, and acquisition time = 3 min. MP2RAGE sequence produced simultaneously three contrast images (INV1, INV2, UNI) and a quantitative T1-mapping (Marques et al., 2010).

### Image processing

A brain morphometry analysis was integrated into the prototype MP2RAGE sequence, providing tissue classification and morphometric segmentation results (Schmitter et al., 2014; Boto et al., 2017). In short, MP2RAGE-UNI image was used to segment the brain into 48 areas, among them. After registering the perfusion-weighted images to the MP2RAGE-UNI images, the obtained 48 brain area masks were used to extract CBF and T1 values for regional comparison between the AD and NC groups. Previous studies have shown that decreased CBF and elevated T1 values in patients with AD occur in specific brain regions (Wolk and Detre, 2012; Wang et al., 2013; Chandra et al., 2019). Therefore, we selected the bilateral thalamus, caudate nucleus, putamen, pallidum, hippocampus, frontal gray matter, parietal gray matter, occipital gray matter, temporal gray matter, cingulate gray matter, corpus callosum white matter, and insula as regions of interest to reduce the overfitting problem caused by irrelevant brain regions.

### Statistical analysis

All data were analyzed using SPSS (version 24.0),^1^ MedCalc 19.0, and MATLAB (MathWorks, Natick, MA, United States). The Kolmogorov–Smirnov and Levene tests were used to assess the normality and variance of CBF and T1 values. Data conforming to a normal distribution are expressed as ${\text{x}}^{-}$ ± s, and non-conformities are denoted by M (Q1, Q3). Normally distributed data with homogeneous variances were compared between AD and NC groups using independent samples *t*-tests, and those with non-homogeneous variances used corrected *t*-tests. The non-parametric Mann–Whitney U test was used for those that were not normally distributed. The gender difference between the two groups was tested by chi-square. Logistic regression analysis was used to identify brain regions associated with AD diagnosis and establish a combined model. The diagnostic efficacy of each brain region and the combined model for AD were assessed using receiver operating characteristic (ROC) curves, and differences among non-homogeneous variances the areas under the curve (AUCs) were compared using the DeLong method. The correlation between MMSE scores was analyzed using partial correlation analysis, and the level of significance was set at *P* < 0.05. Multiple comparisons were controlled using false discovery rate (FDR) correction (corrected *p* < 0.01).

## Results

**FIGURE 1:**
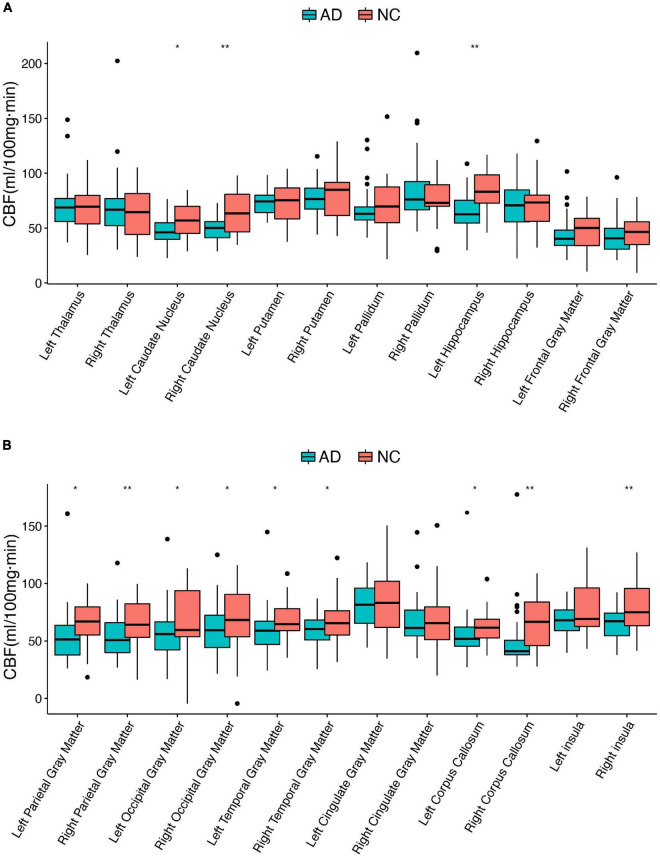
Differential cerebral blood flow between AD and NC individuals in 24 brain regions. AD, Alzheimer’s disease; CBF, cerebral blood flow; NC, normal controls; *corrected *p* < 0.05; **corrected *p* < 0.01. **(A,B)** Boxplots of the CBF in different brain regions.

Demographics of the 78 participants are shown in Table 1. The differential CBF in different brain regions between AD and NC individuals is shown in Figure 1. There were five brain regions with significantly lower CBF values in patients with AD compared with the NC subjects, including the right caudate nucleus, left hippocampus, right parietal gray matter, right corpus callosum, and right insula. Comparing the T1 values of the above five brain regions using the same method, the right caudate nucleus, left hippocampus, right parietal gray matter, and right insula were also significantly different (corrected *p* < 0.01). The T1 value of the right corpus callosum was higher in the NC group, but the difference was not statistically significant (corrected *p* = 0.375).

**TABLE 1:** Demographic and clinical data.

	AD group	NC group	*p*
Number	45	33	–
Age (years)	73.51 ± 7.51	70.51 ± 7.88	0.092
Gender (female %)	64.44	63.64	0.941
BMI	22.59 ± 2.70	23.49 ± 2.57	0.142
Educational years	10.82 ± 3.41	10.70 ± 3.50	0.874
MMSE	18.38 ± 3.07	27.12 ± 1.34	<0.001

AD, Alzheimer’s disease; BMI, body mass index; MMSE, Chinese versions Mini-mental State Examination; NC, normal control.

**FIGURE 2:**
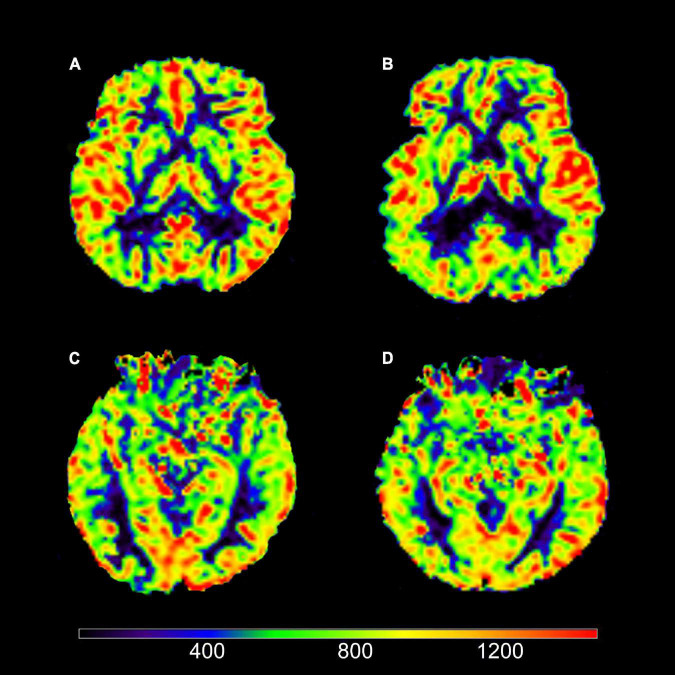
Axial view of cerebral blood flow (CBF). **(A)** Image of a typical AD patient (female; 67 years old). **(B)** Image of a NC subject (male; 73 years old). **(C)** Image of a typical AD patient (female; 64 years old). **(D)** Image of a NC subject (female; 64 years old). It could be intuitively shown that **(A)** views had decreased CBF in right caudate nucleus (RCc) compared to **(B)** views; **(C)** views had decreased CBF in left hippocampus (LHc) compared to **(D)** views.

According to logistic regression analysis, the right caudate nucleus (odds ratio [OR] 0.959, *p* < 0.05) and left hippocampus (OR 0.960, *p* < 0.05) were the two relevant brain regions for perfusion abnormalities in AD, CBF of the right caudate nucleus (RCc) and left hippocampus (LHc) were show in Figure 2. Then, a multiple combined model was established corresponding to the T1 values.

**FIGURE 3:**
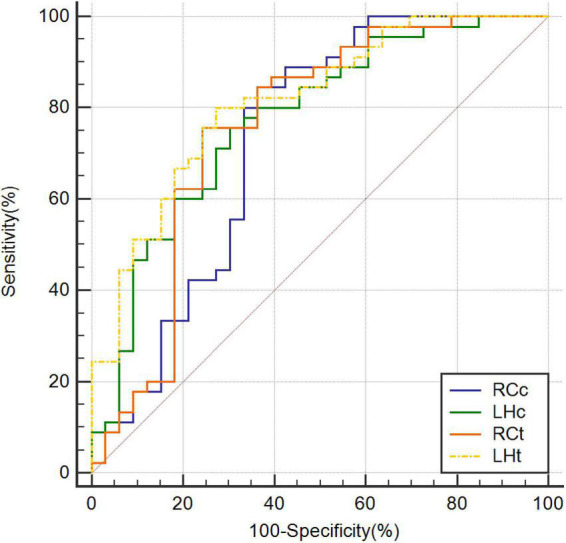
Receiver operating characteristic curves based on CBF of RCc, CBF of LHc, T1 value of RCt, and T1 value of LHt, respectively. CBF, cerebral blood flow; LHc, left hippocampus; LHt, left hippocampus; ROC, receiver operating characteristic; RCc, right caudate nucleus; RCt, right caudate nucleus.

**FIGURE 4:**
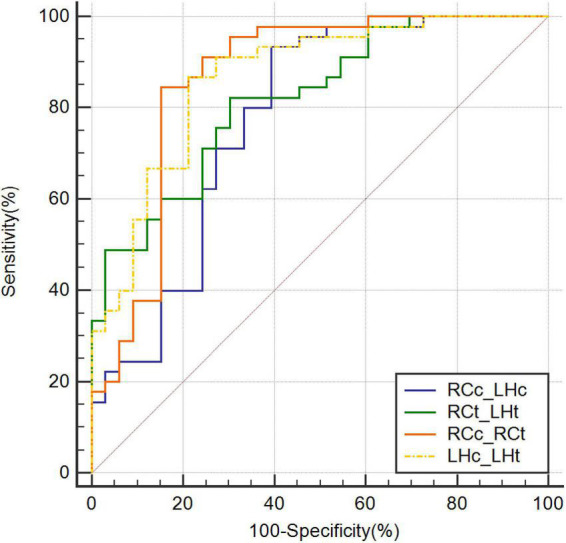
Receiver operating characteristic curves combining two parameters. CBF, cerebral blood flow; LHc_LHt, CBF of left hippocampus and T1 value of left hippocampus; RCc_LHc, CBF of right caudate nucleus and CBF of left hippocampus; RCc_RCt, CBF of right caudate nucleus and and T1 value of right caudate nucleus; RCt_LHt, T1 value of right caudate nucleus and T1 value of left hippocampus; ROC, receiver operating characteristic.

**FIGURE 5:**
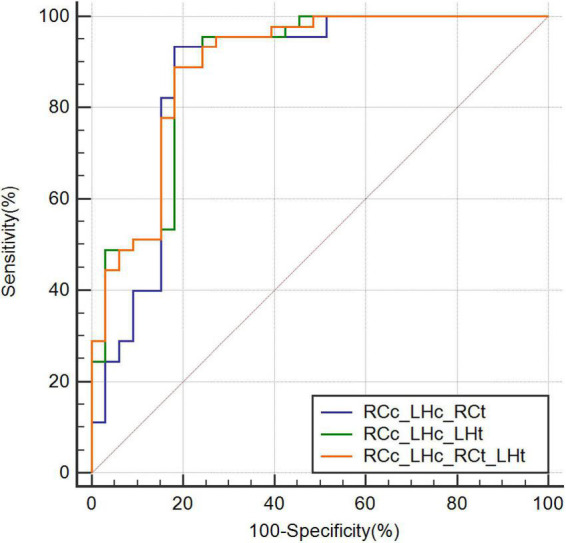
Receiver operating characteristic curves combining three or more parameters. CBF, cerebral blood flow; RCc_LHc_LHt, CBF of right caudate nucleus and, CBF of left hippocampus, and T1 value of left hippocampus; RCc_LHc_RCt, CBF of right caudate nucleus and, CBF of left hippocampus, and T1 value of right caudate nucleus; RCc_LHc_RCt_LHt, CBF of right caudate nucleus and, CBF of left hippocampus, T1 value of right caudate nucleus, and T1 value of left hippocampus; ROC, receiver operating characteristic.

The highest AUC value was 0.894 (sensitivity = 0.889, specificity = 0.818), obtained by combining CBF in the right caudate nucleus (RCc), CBF in the left hippocampus (LHc), T1 value in the right caudate nucleus (RCt), and T1 value in the left hippocampus (LHt) (Figures 3–5 and Table 2). On DeLong inspection, there was no significant difference in the diagnostic efficacy between the four single-parameter diagnostic models. The combined model had different degrees of increased diagnostic efficacy compared with the single-parameter model. RCc compared with RCc_LHc_RCt_LHt (*p* = 0.001); LHc compared with RCc_LHc_RCt_LHt (*p* = 0.006); RCt compared with RCc_LHc_RCt_LHt (*p* = 0.023); and LHt compared with RCc_LHc_RCt_LHt (*p* = 0.066).

**TABLE 2:** Receiver operating characteristic (ROC) curves of various parameter combinations.

	Cut-off	Sensitivity	Specificity	Youden index	AUC	
					Mean	95% CI
RCc	59.902	0.844	0.636	0.480	0.731	0.609–0.854
LHc	76.808	0.756	0.697	0.453	0.772	0.666–0.879
RCt	1191.832	0.756	0.758	0.513	0.759	0.641–0.0877
LHt	1320.708	0.800	0.727	0.527	0.813	0.718–0.908
RCc_LHc	0.457(RCc = 63.394, LHc = 77.987)	0.933	0.606	0.539	0.775	0.664–0.886
RCt_LHt	0.557(RCt = 1309.103, LHt = 1301.550)	0.822	0.697	0.519	0.820	0.729–0.910
RCc_RCt	0.556(RCc = 60.295, RCt = 1191.832)	0.844	0.849	0.693	0.865	0.772–0.957
LHc_LHt	0.491(LHc = 85.468, LHt = 1311.493)	0.867	0.788	0.655	0.866	0.784–0.948
RCc_LHc_RCt	0.424(RCc = 65.090, LHc = 83.520, RCt = 1191.682)	0.933	0.812	0.752	0.871	0.770–0.962
RCc_LHc_LHt	0.329(RCc = 65.090, LHc = 83.520, LHt = 1281.999)	0.956	0.758	0.713	0.887	0.809–0.965
RCc_LHc_RCt_LHt	0.452(RCc = 47.451; LHc = 83.771; RCt = 979.333; LHt = 1260.170)	0.889	0.818	0.707	0.894	0.819–0.968

AUC, area under the curve; CBF, cerebral blood flow; LHc, CBF of left hippocampus; LHt, T1 value of left hippocampus; RCC, CBF of right caudate nucleus; RCt, T1 value of right caudate nucleus.

In the entire cohort of the AD and NC groups, excluding the interference of sex, age, years of education, and body mass index (BMI), RCc, LHc, RCt, and LHt were statistically significantly correlated with MMSE scores through partial correlation analysis, and the correlation cofficient of LHc is higher (*r* = 0.572, *p* < 0.01) (Table 3).

**TABLE 3:** Clinically observed severity measurements.

		RCc	LHc	RCt	LHt
MMSE	r	0.469	0.578	−0.236	−0.456
	p	<0.001	<0.001	0.043	<0.001

LHc, CBF in left hippocampus; LHt, T1 value in left hippocampus; MMSE, Chinese versions Mini-mental State Examination; RCc, CBF in right caudate nucleus; RCt, T1 value in right caudate nucleus.

## Discussion

This study investigated the diagnostic value of multi-modality quantitative MRI parameters. To the best of our knowledge, this is the first demonstration of the diagnostic power of CBF combined with T1-mapping. Group comparison found lower CBF values and higher T1 values in several brain areas in patients with AD compared with the NC group. Further ROC analysis demonstrated that a combined model based on both quantitative parameters achieved better diagnostic performance than either single parameter.

The present cohort included 45 AD and 33 NC subjects and compared CBF in 24 brain regions. Apart from the right thalamus and right pallidum, the mean perfusion values were reduced in the remaining 22 brain regions of patients with AD. According to previous AD neuroimaging studies, the pattern of reduced CBF in patients with AD is primarily concentrated in the hippocampus, basal nuclei clusters, and cognitive correlation cortical gray matter. Our study identified five brain regions (right caudate nucleus, left hippocampus, right parietal gray matter, right corpus callosum, and right insula) with significant differences when corrected by multiple comparisons (FDR correction, *p* < 0.01), consistent with findings from previous studies (Wolk and Detre, 2012; Wang et al., 2013; Wang, 2016; Camargo et al., 2021; Duan et al., 2021). These regions are closely associated with the development of AD. The caudate nucleus is a gray matter mass embedded in the medulla, buried deep in the base of the brain, responsible for the fine-tuning and coordination of movements (Valera-Bermejo et al., 2021). The hippocampus is a memory and cognitive center and is related to the occurrence and progression of AD (Dautricourt et al., 2021). The association between the corpus callosum and insula in AD is unclear, and some theories remain controversial and contradictory (Kamal et al., 2021; Giacomucci et al., 2022). However, the mean CBF values in the right thalamus and pallidum were elevated in the AD group, but these differences were not significant (*p* > 0.05). This result is similar to the previous finding of increased CBF in the basal nucleus cluster in the pre-AD period (Hays et al., 2018). Although the brain regions found in the Hays et al. study are not consistent with previous studies, the authors suggest that the reason for this change is suggestive of neurodegeneration leading to CBF dysregulation and the existence of a neural compensatory mechanism for cognitive decline in some brain regions.

T1-mapping has been used to study many central nervous system disorders such as epilepsy (Massire et al., 2021), multiple sclerosis (Massire et al., 2021; Thaler et al., 2021) and depression (Li et al., 2019). However, the findings of AD research are controversial (Tang et al., 2018). Su et al. found that T1 values in patients with AD at baseline were reduced in temporal and parietal lobes, which is contrary to our results. Interestingly, in the original study, as the disease worsened, T1 values increased significantly in the right caudate, bilateral hippocampus, and other regions instead of decreasing, indirectly complementing our findings (Su et al., 2016). An earlier classical low-magnetic field MRI-based study showed an overall trend in T1 values that was consistent with our results, but they did not precisely stage the brain (Besson et al., 1989). The application of high field strength in our study increased the image signal-to-noise ratio, making the image segmentation more detailed and study results more reliable. Pelkmans et al. reported that myelin determines the conduction of neuronal signals along axonal connections in brain networks, and loss of myelin integrity might result in cognitive decline in AD. Changes in T1 values can reflect myelin content, and myelin is generally associated with increased T1 values (Pelkmans et al., 2019). Future research should further investigate the predicted value of T1-mapping.

In our study, T1 values in the right caudate nucleus and left hippocampus demonstrated good diagnostic performance (AUC = 0.759 and 0.813, respectively). We also combined CBF and T1 values, which improved the diagnostic efficacy compared with that of each single parameter. The highest achievable AUC of 0.894 (sensitivity = 0.889, specificity = 0.818) was obtained using a combination of four parameters. The combination of CBF and T1 values also improved the diagnostic rate (AUC = 0.775–0.865). Our multiparametric MRI measurements improved the results for AD discrimination from NC. Furthermore, several previous studies determined cutoff values in a relatively subjective manner (e.g., less than twice the standard deviation of the control group mean) (Raji et al., 2010). In this study, we used the Jorden index, which together reflects sensitivity and specificity, as a scientific measure to derive the optimal cutoff value for our local cohort.

Like previous studies (Duan et al., 2021; Giacomucci et al., 2022), in our cohort, the severity of neuropsychologic impairment was strongly associated with brain scan measurements. A previous study confirmed that gender, age, education level, and BMI affected cognitive decline (Nebel et al., 2018); therefore, we performed a partial correlation analysis to exclude multiple confounding factors. RCc, LHc, and LHt were found to be powerful predictors of the clinically observed severity measurements.

There are several limitations of this study. First and most importantly, multiple stages of progressive diseases such as subjective cognitive decline and mild cognitive impairment, were not included. Second, we only performed cross-sectional diagnosis in all patients; future longitudinal analysis is needed. Long-term clinical follow-up may improve the diagnostic accuracy for AD. Third, the superiority of temporal lobe diagnosis was not demonstrated in our cohort (Alexopoulos et al., 2012). The temporal lobe, especially the medial temporal lobe, is associated with memory consolidation. This discrepancy may be due to the small amount of data we had and possibly to the fact that the pCASL scanning technique still needs to be refined. Future large-scale clinical studies to validate the diagnostic accuracy and robustness of the CBF and T1 values are imperative.

## Conclusion

In conclusion, combining the pCASL and T1-mapping methods is superior to using a single measure in discriminating AD and NC cohorts. T1-mapping is a competitive technique that provides quantitative measurements of pathologic changes in the brain. A “one-stop-shop” study of multimodal parameters in the future is essential for diagnosing and monitoring AD.

## Data availability statement

The datasets presented in this study can be found in online repositories. The names of the repository/repositories and accession number(s) can be found in the article/supplementary material.

## Ethics statement

The studies involving human participants were reviewed and approved by the Ethics Committee of Beijing Hospital. The patients/participants provided their written informed consent to participate in this study. Written informed consent was obtained from the individual(s) for the publication of any potentially identifiable images or data included in this article.

## Author contributions

XW, CL, and MC designed the work. XW, DW, and XL collected and integrated the data. XW, WW, PG, and BL analyzed the data and prepared the manuscript. JP, XZ, and JZ edited and revised the manuscript. All authors contributed to the article and approved the final manuscript.

## Footnotes

1
http://www.spss.com

## Abbreviations

AD: Alzheimer’s disease
ASL: arterial spin labeling
AUC: area under the curve
CBF: cerebral blood flow
FDR: false discovery rate
LHc: CBF in the left hippocampus
LHt: T1 values in the left hippocampus
MMSE: Mini-Mental State Examination
MP2RAGE: magnetization-prepared 2 rapid acquisition gradient echo images
MRI: magnetic resonance imaging
pCASL: pseudo-continuous ASL
RCc: CBF in the right caudate nucleus
RCt: T1 values in the right caudate nucleus.
